# Revealing selection in cancer using the predicted functional impact of cancer mutations. Application to nomination of cancer drivers

**DOI:** 10.1186/1471-2164-14-S3-S8

**Published:** 2013-05-28

**Authors:** B Reva

**Affiliations:** 1 Computational Biology Center, Memorial Sloan-Kettering Cancer Center, 1275 York Avenue, NY 10065, USA

## Abstract

Every malignant tumor has a unique spectrum of genomic alterations including numerous protein mutations. There are also hundreds of personal germline variants to be taken into account. The combinatorial diversity of potential cancer-driving events limits the applicability of statistical methods to determine tumor-specific "driver" alterations among an overwhelming majority of "passengers". An alternative approach to determining driver mutations is to assess the functional impact of mutations in a given tumor and predict drivers based on a numerical value of the mutation impact in a particular context of genomic alterations.

Recently, we introduced a functional impact score, which assesses the mutation impact by the value of entropic disordering of the evolutionary conservation patterns in proteins. The functional impact score separates disease-associated variants from benign polymorphisms with an accuracy of ~80%. Can the score be used to identify functionally important non-recurrent cancer-driver mutations? Assuming that cancer-drivers are positively selected in tumor evolution, we investigated how the functional impact score correlates with key features of natural selection in cancer, such as the non-uniformity of distribution of mutations, the frequency of affected tumor suppressors and oncogenes, the frequency of concurrent alterations in regions of heterozygous deletions and copy gain; as a control, we used presumably non-selected silent mutations. Using mutations of six cancers studied in TCGA projects, we found that predicted high-scoring functional mutations as well as truncating mutations tend to be evolutionarily selected as compared to low-scoring and silent mutations. This result justifies prediction of mutations-drivers using a shorter list of predicted high-scoring functional mutations, rather than the "long tail" of all mutations.

## Introduction

Numerous somatic mutations are detected in thousands of genes in all cancers [1-13]. Mutations vary in their impact on a gene's function [14,15] and in their contribution to cancer [16-18]. Every tumor has its own mutation spectrum of ~10 to 10,000 of protein-altering mutations. A challenge is to identify mutations that provide a selective advantage to tumors ("drivers"). Knowing driver mutations for individual tumors, one can develop the personalized approaches to treat cancer [19].

Driver mutations are commonly determined from distributions of mutations in a large group of tumor samples [1,20-24]. It is assumed that many of the tumors are under similar selection pressure and those mutations, which are fixed more frequently than expected based on a given background mutation rate (e.g. recurrent mutations observed in many tumors and across many cancers [25]) give selective advantage to cancer. It is also assumed (although rarely articulated) that the number of cancer-causing combinations of driver mutations is limited and therefore a large enough set of sequenced cancer genomes will represent all combinations of driver mutations in an amount sufficient for statistical conclusions.

However, massive sequencing of cancer genomes [1-13] has revealed an enormous diversity of genomic aberrations as well as the high diversity of background mutation rates within many types of common cancers [8,9]. The huge diversity of genomic alterations and mutation rates obviously limits the predictive power of statistical approaches. Typically, genomic alterations in the top cancer genes found by statistics do not affect all tumors [1-7,10-13]. Thus, statistical approaches leave two important questions without answers: First, are there more genes contributing to carcinogenesis in a given type of cancer? Second, what are the concrete driver mutations in a given tumor?

An alternative, personalized approach is to determine cancer drivers based on in-depth analysis of the impact a mutation may have on protein molecular function in the tumor-specific context of genomic alterations. Currently, the implementation of this approach as a primary method for determining drivers is limited by incompleteness of the present knowledge of gene function and gene-regulation networks, and insufficiency of the existing molecular modeling approaches. Typically, the assessment of the functional impact of mutations is used in the subsequent analysis of already found driver mutations [12,13,26-28]. However, more accurate predictions of driver mutations can be achieved by integration of the statistical and the functional approaches. Hence, new approaches have been recently reported [13,29], which integrate functional predictions and mutation distribution statistics. However, the methodology of integration of statistical and functional information is not yet well established. In particular, the statistical model of [29] is not applicable for determining drivers in individual tumors; it is also unclear what is the actual power of the "functional mutation burden" [13] to predict driver mutations.

Recently, we introduced the functional impact score (FIS), which assesses the functional impact of a mutation by a value of entropic disordering of the evolutionary conservation patterns in protein families and subfamilies [30]. The FIS function (implemented as a web-based service mutationassessor.org) was validated by assessing the accuracy of separation of known disease-associated variants from benign polymorphisms and by separation of known recurrent cancer mutations (drivers) from single mutations (passengers) [25,31]. The original FIS function of the mutation assessor was also independently tested and integrated with other mutation scores in the CONDEL [32] and Oncodrive-FM [29] methods; the FIS function was recently implemented and rigorously tested in the "transFIC" approach to differentiate driver and passenger mutations [33].

However the fact that the FIS of the mutation assessor (or other approaches) differentiates preselected drivers from passengers does not automatically mean that it will not produce too many false positives in analysis of total sets of somatic mutations found in tumors. Therefore, before using the FIS to nominate driver mutations in a large set of somatic mutations, it is necessary to answer an important practical question: how the value of the predicted functional impact correlates with *the contribution of a given mutation to carcinogenesis? *Assuming that cancer-drivers are positively selected in tumor evolution, we propose and test a hypothesis: "*high scoring functional mutations tend to be selected in tumor evolution*". Testing this hypothesis is interesting because the FIS represents the evolutionary conservation of residues; a value of the score can be simply interpreted as a measure of conservation. Testing this hypothesis is also practical because the impact score of the mutation assessor is used routinely for assessment of the mutation impact in large-scale sequencing projects [3-6,11,12] and in newly developed combined approaches [29,32,33].

This hypothesis has several testable implications. If it is true, then the fraction of cancer genes (e.g. tumor suppressors and oncogenes) should increase among genes affected by functional mutations. Another general signature of selection, non-uniformity of distribution of mutations across genes, should also increase among functional mutations. Functional mutations should more frequently affect genes, which are likely under selection pressure, i.e. genes affected by truncating mutations or by copy number alterations.

Therefore, we tested the hypothesis by comparing distributions of silent, truncating and missense mutations categorized by the predicted functional impact [30]. We investigated how the predicted functional impact correlates with the frequency of affected tumor suppressors and oncogenes, non-uniformity of distribution of mutations and frequency of concurrent genomic alterations. These tests are general and can be used in studying selection and nominating driver mutations using any scoring function.

All tests conducted on ~120K missense mutations among six types of cancers studied by TCGA showed that high-scoring functional mutations tend to be evolutionary selected. These results justify nominations of the driver mutations based on the predicted functional impact score of the mutation assessor.

## Results and discussion

Cancer-driver mutations are defined as those that give selective advantage to cancer cells. Therefore cancer-driver mutations are specifically selected in tumor evolution. It is easy to identify as evolutionarily selected recurrent cancer mutations. The distributions of the FIS for recurrent mutations and disease-associated variants are practically indistinguishable [30]. Can the FIS be used to bring on the top both recurrent and non-recurrent cancer-driver mutations? To this end, one needs to prove that non-recurrent high-scoring mutations are generally under stronger selection pressure as compared to low-scoring or silent mutations. Below we present computational tests that reveal a stronger selection pressure for predicted high-scoring functional mutations.

**Figure 1:**
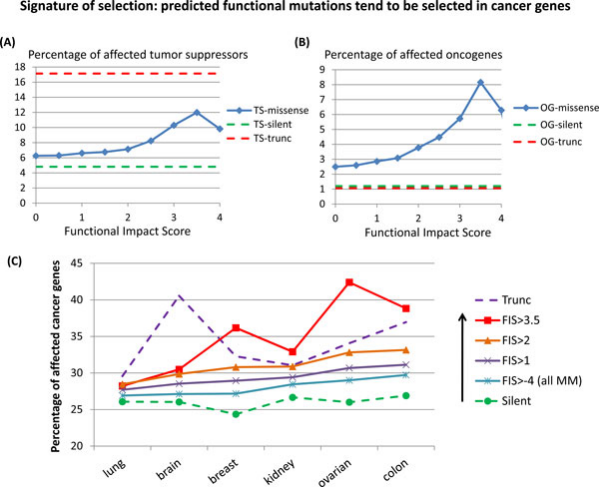
**(A,B) Percentages of predicted functional missense mutations in annotated tumor suppressors (TS) (A) and oncogenes (OG) (B) tend to increase with the value of the FIS in colon cancer **[4]. Percentages of "silent" and "truncating" mutations are given for comparison; "TS-missense", "TS-silent", TS-trunc" stand for annotated tumor suppressors affected by respectively, missense, silent and truncating mutations; similarly, OG-missense", "OG-silent", OG-trunc" stand for annotated oncogenes affected by respectively, missense, silent and truncating mutations; **(C) **Percentage of annotated cancer genes affected by missense mutations tend to *increase *with the predicted functional impact for missense mutations detected in each of six TCGA projects [3-6,10,11]. All missense mutations are separated into 4 groups by a value of the predicted functional impact; thus, "FIS>-4 (all MM)" stands for a mutation group that includes all assessed missense mutations (MM); "FIS>1" stands for a mutation group that includes all mutations assessed with FIS>1, etc... Percentages of "silent" and "truncating" mutations affecting annotated cancer genes in six types of studied cancers are given for comparison.

First, we studied how a fraction of "cancer genes" (tumor suppressors and oncogenes) affected by missense mutations depends on the value of the functional impact score. We tested and confirmed a hypothesis that the fraction of cancer genes affected by mutations increases with the value of the functional impact score (Figure 1).

Figures 1A and 1B present distributions of truncating (TM), silent (SM) and predicted functional missense mutations (FM) affecting tumor suppressors and oncogenes in colon cancer 4. (The lists of tumor suppressors (TS) and oncogenes (OG) are taken from the annotated lists of cancer genes (Additional File 1, Tables S1; Additional File 2[30,34,35]).

In spite of the fact that the cancer gene list is incomplete, non-specific to a given cancer and have erroneous annotations, the distributions of truncating, silent and predicted functional mutations clearly demonstrate natural selection. First, one should note a striking difference between truncating mutations and silent mutations affecting tumor suppressors (Figure 1A) and oncogenes (Figure 1B). Truncating mutations affect tumor-suppressors approximately three times more often than silent mutations, while they affect oncogenes with the same frequency as silent mutations. The difference in frequencies is caused by natural selection. Truncating mutations result in loss of function of certain tumor suppressors that give advantage to affected cancer cell. Therefore truncating mutations in tumor suppressors are been fixed in evolution. However, truncating mutations in oncogenes are not generally advantageous to cancer cells, and, hence, they are not fixed in tumor evolution. The distributions of predicted functional missense mutations also show the clear tendency of high-scoring mutations to be evolutionarily selected for tumor suppressors and oncogenes as compared to low-scoring and silent mutations (Figure 1A-B). With an increase of the functional impact (FIS), a fraction of mutations affecting tumor suppressors and oncogenes increases and gets the maximum value at FIS ~3.0. At higher values of FIS, the total number of mutations becomes very low that may affect statistics.

The Figure 1C presents distributions of silent, truncating and predicted functional mutations affecting all annotated cancer genes (Additional File 1, Table S1; Additional File 2) in several cancer types (TCGA). While the fractions of silent mutations affecting cancer genes, stays about the same across all studied cancers, the fractions of truncating and predicted functional mutations vary significantly for different cancers. What is the most remarkable is that the fractions of affected cancer genes increase with the value of the functional impact score for all cancers, i.e. predicted functional mutations tend to be selected in cancer genes in different type of cancers.

However, the observed shift of the FIS distribution of mutations in cancer genes towards higher values can be also explained by better evolutionarily conservation of cancer genes [36]. (Let's assume that cancer genes are conserved significantly better than non-cancer genes. Then, uniformly (or randomly) distributed mutations in cancer genes will automatically get higher FIS values and a fraction of cancer genes will be disproportionally high among high-scoring mutations. Under this assumption, the observed enrichment of high-scoring mutations in cancer genes (Figure 1) will simply reflect the better conservation of cancer genes, rather than selection of the specific mutations in cancer genes).

Selection of mutation in tumor evolution results in non-uniformity of mutation distributions. The non-uniformity of mutation distributions is especially high in cancer genes, many of which are affected by recurrent mutations. Therefore, to assess an applicability of the FIS to predict driver mutations, one needs to answer a key question: what is a correlation between the value of the FIS and the non-uniformity of mutation distribution? This question is based on the following hypothesis: driver mutations are selected in special (and therefore better conserved) positions of cancer genes and scoring higher than passenger mutations. Then, the higher the score, the more likely the mutation is a driver, and the distribution of high scoring mutations should reflect the main feature of selection - more mutations in fewer genes. The alternative hypothesis is that driver and passenger mutations in cancer genes are scoring essentially equally. Then, the FIS is not relevant for differentiating drivers and passengers. Thus, the question of what factor plays the major role in the increase of a fraction of high-scoring mutations in cancer genes - the better conservation of cancer genes in evolution of species or the specific selection of driver mutations in tumor evolution - is actually superseded by other questions: does the non-uniformity of mutation distribution increase with the value of the FIS, and, does the non-uniformity of distribution increase for high-scoring mutations in cancer genes (many of which are under selection pressure) versus non-cancer genes (many of which are not under selection)?

**Figure 2:**
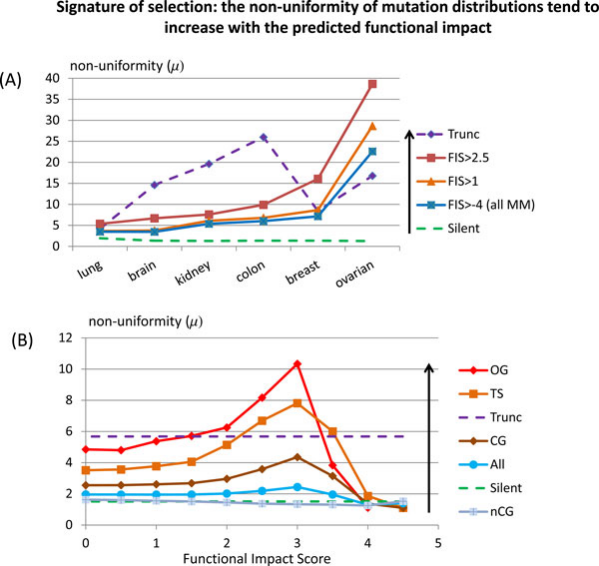
**(A) The non-uniformity of distributions of mutations across annotated genes-tumor suppressors increases with a value of the predicted functional impact for missenese mutations discovered in each of six TCGA projects **[3-6,10,11]; **missense mutations are separated into three groups by the predicted functional impact**. The non-uniformity, ***μ***, is defined as a ratio of the total number of affected genes in a dataset to the "effective" number of genes that carry the majority of mutations (Eq.2); ***μ~1 ***means that mutations are distributed fairly uniformly across genes; ***μ≫1 ***means that majority of mutations are **selected **in a small fraction of all mutated genes. The non-uniformities of "Silent" and truncating mutations ("Trunc") affecting the same groups of genes are given for comparison. (B). The non-uniformities of mutation distributions computed for different gene groups in glioblastoma (brain) cancer [6]. "All" stands for mutations affecting all genes; TS, OG, CG, nCG, stand for mutations affecting, respectively, annotated tumor-suppressors, annotated oncogenes, annotated cancer genes, genes with no cancer annotations; the non-uniformities of truncating ("Trunc") and "Silent" mutations are computed for truncating and silent mutations affecting all genes.

To answer these questions, we introduced the numerical indicator of the "non-uniformity" of mutation distribution across genes and used it as a measure of selection of somatic mutations in cancer. The non-uniformity can be numerically determined as a ratio of the total number of mutated genes to the effective number of mutated genes that carry majority of mutation (Eq.2, Methods). The higher this ratio, the higher the non-uniformity. (The non-uniformity of a distribution does not depend on the score, therefore any non-specific bias (shift) of the FIS distribution within a given group of genes (e.g. potential shift of the FIS caused by better conservation of cancer genes) does not affect the non-uniformity). The non-uniformities of distributions of truncating, silent, missense and predicted functional mutations computed for different types of cancer are presented in Figure 2.

As expected, distributions of predicted functional mutations and truncating mutations are essentially non-uniform (μ~5-40) that differ them drastically from the more uniform distributions of silent mutations (μ~1.4-1.9). The non-uniformity of distributions increases with the value of the functional impact showing the increase of selection pressure for predicted functional mutations (Figure 2A).

We also compared the non-uniformities of distributions of predicted functional mutations affecting different groups of genes. The non-uniformities of distributions were computed for predicted functional mutations affecting all genes, annotated cancer genes, annotated tumor suppressors and oncogenes, and, genes that have no cancer annotations. The Figure 2B presents typical dependencies obtained for glioblastoma cancer. Similar results are obtained for all studied cancers. The non-uniformity of distributions in cancer genes increases with an increase of predicted functional impact, while the non-uniformity of the mutation distribution in non-cancer genes does not increase and even has a tendency to decrease (the lower μ, the bigger non-uniformity). The non-uniformity μ gets the maximal value at FIS ~3.0 and starts to decrease at higher FIS. This simply reflects the drastic decrease of a number of mutations and a number of affected genes at higher FIS.

Computing non-uniformity of distributions, we did not take into account gene length. Differences in gene lengths can affect computed values of non-uniformity, especially when the number of genes and mutations are small and differences in gene lengths are big. Although, in the general case, the non-uniformity of a mutation distribution may depend upon a spectrum of nucleotide substitution and cancer type, the main effect of gene length differences can be assessed by assuming that mutations are distributed proportionally to gene lengths. Thus, we determined the effective number of genes that would carry majority of uniformly distributed mutations. The coding length of human genes was taken from MAPBACK database [37]. We found that the effective number of the longest genes, which cover the whole genome is ~9,400 that gives for the non-uniformity coefficient a value of ~2. Thus, the non-uniformity of the unbiased mutation distribution caused by the difference in gene lengths is very close to the non-uniformity coefficients computed for the observed distribution of silent mutations across different cancers (~1.4-2).

However, taking into account gene lengths is not necessary for comparison characteristics of distributions of the whole mutation classes (truncating, silent, missense) affecting the same large groups of genes (thousands of mutations and genes). The hallmark of selection can be seen in the significant increase of μ from 3.5 to 7.8 for predicted functional mutations affecting tumor suppressors at FIS~3.0; correspondingly, no selection is observed for mutations affecting non-cancer genes or silent mutations. Actually, one can compare non-uniformity of mutation distributions for different groups of genes: if the numbers of mutated genes in gene groups are large enough (~100 or more), the effects of different gene lengths on non-uniformity of distributions become insignificant because of averaging large numbers of mutations affecting genes of different lengths. Therefore the non-uniformity coefficients μ are generally small (close to one) for silent mutations and large for truncating and predicted functional mutations selected in tumor suppressor, oncogenes and all cancer genes.

We report more details comparing the non-uniformity of mutation distributions in cancer genes and in non-cancer genes for high-scoring missense mutations, for all missense mutations, for combination of high-scoring mutations and truncating mutations and for truncating mutations taken alone (Additional File 1, Table S2). The main results of these tests can be summarized as follows: (i) the non-uniformity of distributions of high-scoring functional missense mutations in cancer genes is always higher as compared to the non-uniformity of all missense mutations both in cancer genes and in non-cancer genes; (ii) the non-uniformity of mutations distribution increases for combination of missense mutations and truncating mutations; (iii) the non-uniformity of mutation distributions is the highest for combination of the high-scoring missense mutations and truncating mutations in cancer genes. These results resolve the question of biasing of the FIS caused by potentially better conservation of cancer genes. Regardless of the potential shift of the FIS, the increase of the non-uniformity of distributions of high-scoring mutations in cancer genes proves selection of these mutations in cancer genes.

Thus, the comparison of distributions of missense and predicted functional mutations in combination with truncating mutations both in cancer genes and in non-cancer genes (Figure 1, 2, Additional File 1, Table S2) demonstrates natural selection of predicted high-scoring functional mutations and truncating mutations in cancer genes. Based on this result, one can make recommendations for determining tumor specific (personalized) drivers: nominate as likely drivers high-scoring mutations in known cancer genes; nominate as possible drivers high-scoring mutations in remaining non-cancer genes.

**Figure 3:**
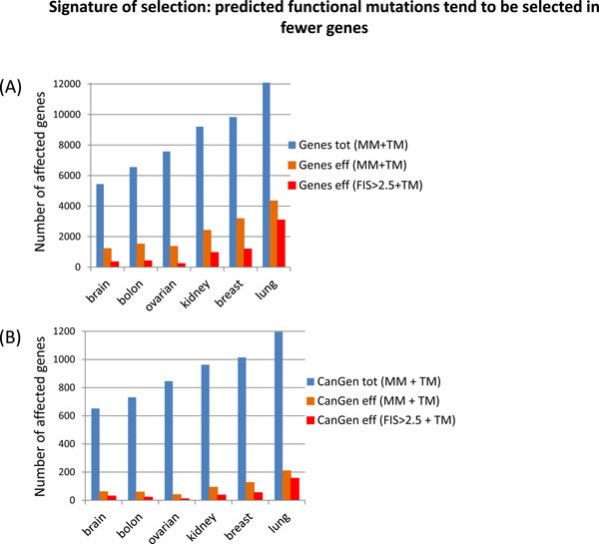
**Histograms of genes affected by all missense mutations and predicted functional mutations built for six types of cancer**: (A) each of the histograms represent the *total *number of genes (Genes tot) affected by at least one of missense or truncating mutations (MM+TM); the *effective *number of genes (Eq.6) affected by at least one of missense or truncating mutations ("Genes eff (MM+TM)"), the *effective *number of genes affected by at least one of predicted functional or truncating mutations ("Genes eff (FIS>2.5+TM)"); (B) the histograms represent the *total *number of cancer genes (CanGene) affected by at least one of missense or truncating mutations (MM+TM); the *effective *number of cancer genes affected by at least one of missense or truncating mutations, the *effective *number of cancer genes affected by at least one of predicted functional or truncating mutations (FIS>2.5+TM).

In Figure 3, we compared the total number of impacted genes and the effective numbers of affected genes (Eqs.3,6) determined for all missense and truncating mutations and for predicted functional and truncating mutations. To determine the effective number of genes impacted by predicted functional mutations, we took all genes impacted by at least one mutation of FIS>2.5, because strong selection of mutations at FIS~2.5-3.0 is visible in all cancers (Figures 1.2). The histograms of Figure 3 show that distributions of mutations across genes are highly non-uniform for all cancers and the non-uniformity increases for predicted functional mutations. The non-uniformity of mutation distributions is higher for cancer genes as compared to all genes. (The actual numbers of genes used in building the histograms are given in Additional File 1, Table S3).

Based on the distributions of Figure 3, one can make estimates of the total numbers of common driver genes for a given cancer. We propose to rank (cancer) genes by a total number of highly functional mutations (FIS>2.5 and truncating mutations) and nominate a set of the "effective genes" as a set of common drivers. This is motivated by the idea that highly functional mutations are selected during tumor evolution in a limited number of conserved positions in certain (cancer) genes. These genes are enriched by highly functional mutations and can be revealed by the increased non-uniformity of distributions of highly functional mutations.

**Figure 4:**
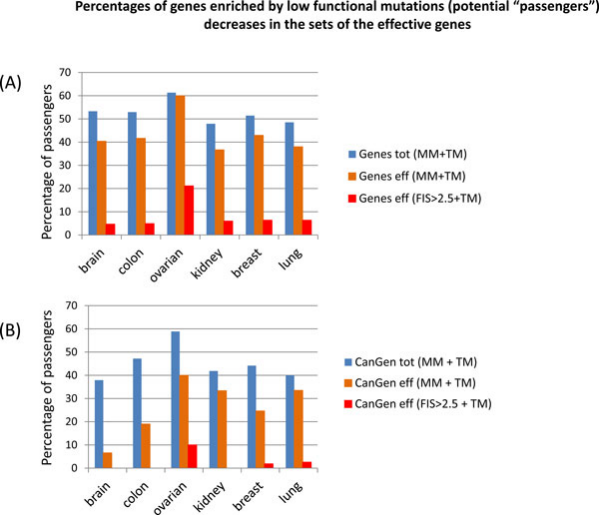
**The percentages of potential "passenger" genes predicted within genes sets presented in the histograms of Figure 3**. Potential "passengers" genes are defined as genes that have more or equal number of low functional mutations (FIS<1.0) as compared to a number of high-functional mutations, i.e. missense mutations of FIS>2.5 and truncating mutations; "MM+TM" refers to genes affected by either missense (MM) or truncating (TM) mutations; CanGenes referes to "cancer genes".

However, the effective gene lists can include long genes, which can be incorrectly nominated as common drivers. Long genes can compete with shorter driver genes in a number of highly functional mutations, because long genes have more chances to accumulate such mutations by random. The simplest solution would be removing long genes (e.g. ~30 genes with the exome length bigger than ~15,000 nucleotides) from the lists of the effective genes or from the total list of all mutated genes. However, selection of mutations and role of long genes in cancer is not fully understood, in particular, because not all long genes are mutated proportionally frequently in all cancers. Therefore, rather than excluding long genes from the lists of the effective genes, we implemented a simple criterion for scoring out potentially false positives ("passenger") genes. Assuming that evolutionarily selected genes have more high-functional mutations than low-functional mutations, we marked genes that have more or equal number of low functional mutations as compared to high-functional mutations as potential "passengers". Predicted functional mutations (FIS>2.5) and truncating mutations were counted as high functional mutations; all missense mutations of the FIS<1.0 were counted as benign or low functional (the FIS thresholds are chosen so that to avoid counting mutations in the range of 1<FIS<2.5, where uncertainty of the predicted functional impact is maximal). The percentages of potential passenger genes within each group of the effective genes are presented in Figure 4 and in Additional File 1, Table S3. This simple approach produced reasonable results: typically, fractions of passenger genes are relatively high (>50%) for both a set of "all genes" and a subset of "cancer genes", however the fractions of potential passenger genes drop in the sets of the effective genes and the reduction of passengers becomes especially significant (<5%) for the sets of the effective genes derived with using the functional predictions. (Potentially passenger genes in the sets of the effective genes are long genes, e.g. FAT1 gene in ovarian cancer is 4,588 residues long).

It is difficult to make accurate comparisons between cancers, because the overall diversity of the observed mutation spectrum depends on a number of samples and stage of cancer, but one can notice that the number of the effective genes representing the mutation spectrum for ovarian, colon and brain cancer is smaller than the numbers of the effective genes for kidney, breast and especially lung cancer. Generally, the smaller the number of the effective genes, the stronger the selection. However, the effective number of genes that are likely under selection pressure is estimated as ~200 for ovarian cancer and ~350-400 for brain and colon cancers. The large numbers of genes affected by predicted evolutionarily selected mutations highlight a diversity of cancer drivers and suggest that a typical tumor has more drivers, rather than few drivers. The large numbers of the effective genes have to be compared with the total number of mutated genes; the resulting reduction in numbers of potential driver genes is ~10-30 times. (For more accurate and comprehensive nomination of driver genes, it is necessary to take into account statistics of gene copy number alterations and gene expressions that is beyond the scope of this study).

**Figure 5:**
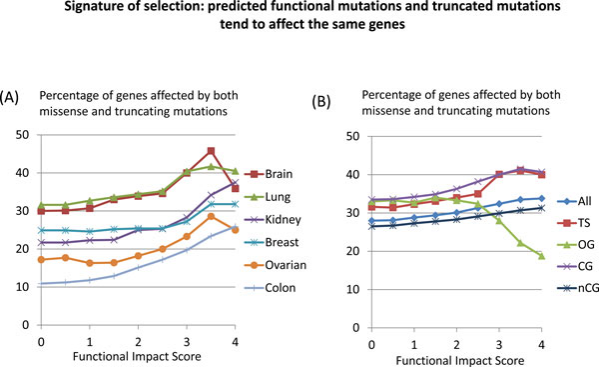
**(A) Percentage of genes tumor-suppressors affected by both predicted functional mutations and truncating mutations increases with the increase of the predicted functional impact of missense mutations discovered in each of six TCGA projects **[3-6,10,11]. **(B) **Percentage of genes affected by both predicted functional mutations and truncating mutations computed for different gene groups in glioblastoma (brain) cancer [6]. "All" stands for mutations affecting all genes; TS, OG, CG, nCG, stand for mutations affecting, respectively, annotated tumor-suppressors, annotated oncogenes, annotated cancer genes, genes with no cancer annotations.

We also studied the statistical concurrency of predicted functional mutations to affect genes that are likely to be under selection pressure. In particular, we considered genes affected by truncating mutations (Figure 5) and genes affected by copy loss or gain (Tables 1, 2). It is reasonable to expect that missense mutations resulting in "loss of function" should be selected more frequently in the same genes, which are affected by truncating mutations. Therefore, a fraction of genes affected by truncating mutations should increase among genes affected by missense mutations of significant functional impact. (It is implied, of course, that each of mutations is detected in a different tumor).

**Table 1:** Percentage of silent (SM) and truncating mutations (TM) affecting genes with different copy number alterations

Cancer		Gene copy number alterations				P-val (-1)	P-val (+1)
				
		-1	0	1	2		
**breast**	CNA:	13	64	21	2		
	SM:	12	65	22	2		
	**TM:**	**17**	64	**18**	2	**<10-6**	**1.0E-06**

**lung**	CNA:	12	63	22	3		
	SM:	11	63	22	3		
	**TM:**	**13**	62	22	3	**2.1E-05**	3.9E-01

**colon**	CNA:	12	71	17	1		
	SM:	11	71	17	1		
	**TM:**	**14**	72	**14**	1	**1.1E-03**	**4.2E-04**

**brain**	CNA:	14	76	8	3		
	SM:	13	76	8	3		
	**TM:**	**15**	75	**7**	2	**9.0E-06**	**4.9E-02**

**kidney**	CNA:	12	76	11	0		
	SM:	10	76	14	1		
	**TM:**	**16**	71	12	0	**<10-6**	1.2E-01

**ovarian**	CNA:	23	51	24	2		
	SM:	21	53	24	2		
	**TM:**	**30**	49	**19**	2	**<10-6**	**<10-6**

Silent mutations are distributed similar to distributions of CNA. Truncating mutations are distributed significantly differently as compared to silent mutations: over-presented in regions of heterozygous deletions; under-presented in regions of copy gains

**Table 2:** Percentage of silent, truncating and functional mutations affecting genes with one copy loss.

Cancer	silent mutation	truncating mutation	P-value	all missense mutations	Missense mutations selected by FIS				
					FIS>2	FIS>2.5	FIS>3.0	FIS>3.5	P-val
**breast**	11.6	**16.7**	0	12.5	12.9	13.4	14.2	**16.3**	2E-06
**lung**	11.4	**13.5**	2E-05	11.7	12.1	12.4	**12.5**	**12.5**	0.009
**colon**	10.7	**13.6**	0.001	11.5	12.4	13.3	14.2	**15.2**	3E-04
**kidney**	6.1	**14.3**	0	6.5	11.3	**12.1**	11.4	10.9	2E-04
**brain**	12.5	**15.3**	9E-06	13.9	14.1	14.6	**15.0**	14.6	0.009
**overian**	20.5	**30**	0	22.3	23.6	26.2	26.0	**29.0**	0

Predicted high-scoring functional missense mutations tend to be selected in genes with one copy loss practically as frequent as truncating mutations.

The data of Figure 5 confirm this expectation. In all studied cancers, fractions of genes - tumor suppressors - affected by both truncating mutations and predicted functional mutations increase at higher values of FIS (Figure 5A). This tendency is general and observed for all genes, but the strongest concurrency between predicted functional mutations truncating mutations is observed for tumor suppressors. The difference in concurrency of predicted functional mutations and truncating mutations affecting different genes groups is well displayed in mutations of lung cancer (Figure 5B). For the total counts of missense mutations, all genes groups have approximately the same percentage of genes affected by truncating mutations. However, among the genes affected by predicted functional mutations, the annotated cancer genes and tumor suppressors are more frequently affected by truncation mutations as compared to the group of "non-cancer genes"; on the contrary, the annotated oncogenes affected by predicted functional mutations are less frequently affected by truncating mutations. These differences demonstrate natural selection of functional mutations in those groups of genes.

Another group of genes, which are likely under selection pressure are genes affected by copy number alterations. Table 1 presents statistics of silent and truncating mutations affecting genes with discretized copy number alterations [38]. Silent mutations with no impact on gene's function (and no selection) are distributed fairly uniformly across genes affected by copy number alterations. Truncating mutations affect protein function; driven by selection, they are distributed significantly differently as compared to silent mutations: over-presented in regions of heterozygous deletions in all studied cancers and under-presented in regions of copy gains (although only in two of six studied cancers).

The percentages of truncating mutations affecting genes with copy loss can be used as a reference for comparison distribution of predicted functional mutations (Table 2).

As expected, predicted functional mutations tend to be selected in genes with copy loss more frequently as compare to silent or low-scoring mutations. Predicted high-scoring functional missense mutations tend to be selected in genes with one copy loss practically as frequent as truncating mutations.

## Method

### The functional impact score

The details of the derivation of the functional impact score of the MutationAssessor are given in [30]. Here we simply review the assumptions used in the derivation. The estimate of the functional impact of a mutation in a given protein sequence is derived from a multiple alignment of homologous sequences under two assumptions: 1) a multiple alignment of protein family sequences is treated as a statistical ensemble at equilibrium; 2) a distribution of residues in any aligned position of a protein alignment is treated independently of other positions in the alignment. In other words, it is assumed that all possible mutations were tried in evolution in each sequence position so that the observed distributions of residues in aligned positions of homologous sequences reflect all possible constraints imposed on these residues. Thus, critically important residues are conserved in the setting of diverse sequence homologs, while evolutionarily unfavorable residues are not observed or observed less frequently than neutral or important residues. In addition to protein family conservation, we use conservation within protein subfamilies, which are derived from clustering multiple sequence alignments [39]. The clustering algorithm groups the sequences of a protein family alignment into distinct subfamilies, so as to minimize the sequence diversity within subfamilies and to maximize the overall difference between subfamilies at a select number of "specificity" positions [39]. Evolutionary constraints are inferred from the patterns of residue conservation in the computed protein subfamilies.

With these assumptions, the mutation impact of a mutation α->β in a sequence position *i *is computed from the observed mutation counts as follows:

(1) $$FIS_{i}\left(\alpha \to \beta \right)=-\frac{1}{2}\left[\text{ln}\frac{n_{i}\left(\beta \right)+1}{n_{i}\left(\alpha \right)}+\text{ln}\frac{n_{{}_{i}}^{p}\left(\beta \right)+1}{n_{{}_{i}}^{p}\left(\alpha \right)}\right]$$

Here *α *and β are residue types (*α, β *= *1,...,21*, indexing *20 *residues types and alignment gaps); $n_{i}\left(\alpha \right)$, $n_{i}\left(\beta \right)$ are, respectively, the numbers of residues of types *α *and *β *in an alignment column *i*; the index *p *refers to the particular subfamily to which the mutated sequence is assigned as the result of clustering and $n_{i}^{p}\left(\alpha \right)$ and $n_{i}^{p}\left(\beta \right)$ are, respectively, the numbers of residues of types α and β in sequence position *i *of a subfamily *p*.

The two terms of Eq.1 are complementary measures of evolutionary conservation; therefore, a combination of these scores provides more information about the potential functional impact of a mutation.

### The statistical measure of the non-uniformity of distributions

Any selection process results in non-uniformity of distributions. Therefore we compared silent, truncating and missense mutations by the non-uniformity of distributions of these mutations across genes. We compared separately the non-uniformity of distributions within different groups of genes such as tumor suppressors (~850), oncogenes (~150), annotated cancer genes (~3,700), and remaining non-cancer genes. We tested a hypothesis that the non-uniformity of distributions increases with the value of the functional impact.

The simple and effective measure of the non-uniformity of a mutation distribution across genes in a given data set can be introduced as a ratio of the total number of mutated genes, Q, to the effective number of mutated genes, K:

(2) μ=Q/K

The effective number of mutated genes in a given dataset, K, is defined as a ratio of the total number of mutations in a data set, M, to the weighted average number of mutations per gene, $\langle N\rangle$,

(3) $$K=M/\langle N\rangle$$

where

(4) $$\langle N\rangle =\sum_{i=1,\ldots ,Q}N_{i}\cdot \left(N_{i}/M\right)=M\sum_{i=1,\ldots ,Q}p_{i}^{2}=M\cdot \lambda$$

and

(5) $$\lambda =\sum_{i=1,...,Q}p_{i}^{2}=\sum_{i=1,...,Q}{\left(N_{i},/,M\right)}^{2}$$

is the Simpson diversity index [40].

Thus,

(6) $$K=M/\langle N\rangle =1/\lambda$$

and

(7) μ=λQ

In the case, when all genes are mutated fairly proportionally $p_{i}=N_{i}/M\sim 1/Q$ that gives $\lambda \sim Q\cdot {\left(1/Q\right)}^{2}=1/Q$. Then the effective number of genes K is close to the actual number of genes K=1/λ~Q and the non-uniformity $\mu =\lambda Q\sim Q\cdot \left(1/Q\right)\sim 1$.

However, when mutations of only one or few genes represent the overwhelming majority of all mutations, the distribution of mutations across genes is extremely non-uniform and the diversity index λ~1. Then the effective number of mutations K=1/λ~1 and μ=λQ~Q becomes a large number, when the total number of genes is a dataset is large.

Thus, the non-uniformity coefficient μ can be used as a measure of selection of mutations in cancer; μ is close to one, when there is no selection or selection is weak and μ is larger, when mutations undergo selection pressure.

### Cancer gene lists

The cancer gene list used in this study is a combination of the three lists: the web-based resource of CancerGenes, which combines gene lists annotated by experts with information from key public databases [35], the cancer genes of Sanger Institute [34] and a gene list of frequently mutated genes with predicted functional mutations [30] derived from the COSMIC database [25,31]. The Additional File 1 (Table S1) provides with summarized statistics in the lists and Additional File 2 presents the actual genes with the basic cancer annotations.

## Conclusion

The main task in analysis of somatic mutations in cancer is determining driver mutations that provide a selective advantage to cancer cells. The recurrence of driver mutations is a signature of selection. Recurrent driver mutations can be differentiated from benign passengers by the predicted functional impact 30. In this work, we showed that the predicted functional impact can be generally applied to identify drivers by revealing trends of evolutionary selection of predicted functional mutations in systematic tests conducted on ~120 missense mutations of six different cancers. We found an important correlation between the value of the predicted functional impact and selection: higher predicted functional impact correlates with stronger selection trends. Hence, we conclude that the functional impact score can be used for prediction of driver mutations and genes.

The functional impact score used in this work [30] represents the evolutionary conservation of residues in protein sequences. The greater values of the score correspond to higher evolutionary conservation. Thus, the conducted tests showed that mutations affecting evolutionary conserved residues tend to be selected in tumor evolution. Or, in other words, rapidly unfolding tumor evolution selects mutations affecting protein residues conserved in millions of years of natural history. This means that the main reservoirs of functional diversity in proteins are the residues that are selected and conserved in molecular evolution.

In this study, we showed that predicted functional mutations (potential drivers) are selected in annotated cancer genes. This underscores the practical usefulness of cancer gene lists. With more cancer genome sequencing, a general list of cancer genes as well as specific cancer gene lists are likely to be very useful in the practice of personalized cancer treatment.

We interpreted as a trend of selection the fact that predicted functional mutations are concurrently selected in genes affected by truncation mutations and by copy number losses. This fact emphasizes the diversity of genomic alterations in cancer. Thus, accurate prediction of cancer driver mutations can be done only in the context of all genomic alterations, possibly by utilizing an integrated profile of functional genomic alterations where predicted functional missense mutation are taken into account together with truncating mutations and gene copy number alterations.

## Competing interests

The author declares that they have no competing interests.

## Supplementary Material

Additional file 1 **contains a Table S1 summarizing the annotations of Cancer Gene List used in the study, a Table S2 presents the non-uniformities of various mutation distributions across six different cancers and a Table S3 presents the effective numbers of genes derived at different thresholds of the FIS**. Click here for file

Additional file 2 **(Table SM2) presents a combined cancer gene list with basic cancer annotations**. Click here for file

## Declarations

The publication costs for this article were funded by NIH grant R01 CA132744-02.

This article has been published as part of *BMC Genomics *Volume 14 Supplement 3, 2013: SNP-SIG 2012: Identification and annotation of SNPs in the context of structure, function, and disease. The full contents of the supplement are available online at http://www.biomedcentral.com/bmcgenomics/supplements/14/S3
