# Supplementary material for: Revealing selection in cancer using the predicted functional impact of cancer mutations. Application to nomination of cancer drivers
Source: BMC Genomics. 2013 May 28;14(Suppl 3):S8. doi: 10.1186/1471-2164-14-S3-S8 (PMC3665576; doi:10.1186/1471-2164-14-S3-S8)
Supplement: Additional file 1 — contains a Table S1 summarizing the annotations of Cancer Gene List used in the study, a Table S2 presents the non-uniformities of various mutation distributions across six different cancers and a Table S3 presents the effective numbers of genes derived at different thresholds of the FIS. [file 1471-2164-14-S3-S8-S1.doc]

Table SI. Review of the combined list of 3761 cancer genes used in the study

| Original gene list | Number of genes |
| --- | --- |
| CBIO MSKCC^1^ | 3164 |
| Sanger^2^ | 454 |
| COSMIC Multiply mutated^3^ | 957 |
| Genes with 1 annotation^1^ | 2536 |
| Genes with >1 annotations | 1225 |
| Genes with >2 annotations | 654 |
| Tumor suppressors | 824 |
| Oncogenes | 152 |
| Tumor suppressors and oncogenes | 42 |

^1^<http://cbio.mskcc.org/CancerGenes/>

^1^Higgins, M.E., Claremont, M., Major, J.E., Sander, C. and Lash, A.E. (2007) **CancerGenes**: a gene selection resource for cancer genome projects. *Nucleic Acids Res*, 35, D721-726.

^2^Futreal, P.A., Coin, L., Marshall, M., Down, T., Hubbard, T., Wooster, R., Rahman, N. and Stratton, M.R. (2004) **A census of human cancer genes**. *Nat Rev Cancer*, **4**, 177-183.

^3^Reva B, Antipin Y, Sander C. Predicting the functional impact of protein mutations: application to cancer genomics. *Nucleic Acids Res*. 2011 Sep 1;39(17):e118.

Table S2. The non-uniformity coefficients µ computed for distributions of missense, predicted functional and truncating mutations in sets of cancer and non-cancer genes.

| **Cancer** | **CG (HFM)** | **non-CG (HFM)** | **CG (MM)** | **non-CG (MM)** | **CG (HFM+TM)** | **non-CG (HFM+TM)** | **CG (MM+TM)** | **non-CG (MM+TM)** | **CG (TM)** | **non-CG (TM)** |
| --- | --- | --- | --- | --- | --- | --- | --- | --- | --- | --- |
| Ovarian | **38.5** | 1.2 | **20.1** | 1.4 | **45.1** | 1.2 | **30.5** | 1.4 | **16.6** | 1.1 |
| Breast | **13.0** | 2.0 | **8.3** | **6.4** | **16.4** | **6.7** | **10.8** | **9.7** | **7.8** | **8.3** |
| Colon | **9.9** | 1.2 | **7.0** | 1.5 | **18.7** | 1.3 | **16.0** | 1.5 | **27.5** | 1.2 |
| Brain | **7.4** | 1.3 | **5.2** | 1.5 | **15.3** | 1.4 | **13.7** | 1.6 | **11.0** | 1.4 |
| Kidney | **6.5** | 1.7 | **4.7** | **4.9** | **20.8** | **3.7** | **14.1** | **6.2** | **19.3** | **3.5** |
| Lung | **5.0** | 1.6 | **3.2** | 1.9 | **6.1** | 1.6 | **3.7** | 1.9 | **3.4** | 1.2 |

The non-uniformity of mutations distributions in cancer genes and in non-cancer genes is computed for missense mutations (MM), predicted high-scoring missense mutations (HFM, FIS>2.5), for truncating mutations (TM), for combination of missense and truncation mutations (MM+TM) and for combination of predicted high-scoring mutations and truncating mutations (HFM+TM). The data of the table show that (i) the non-uniformity of distributions of high-scoring functional missense mutations in cancer genes is always higher as compared to the non-uniformity of all missense mutations both in cancer genes and in non-cancer genes; (ii) the non-uniformity of mutations distribution increases for combination of missense mutations and truncating mutations; (iii) the non-uniformity of mutation distributions is the highest for combination of the high-scoring missense mutations and truncating mutations in cancer genes. These results resolve the question of biasing of the FIS caused by potentially better conservation of cancer genes. Regardless of the potential shift of the FIS, the increase of the non-uniformity of distributions of high-scoring mutations in cancer genes proves selection of these mutations in cancer genes. Thus, high-scoring mutations in known cancer genes (as well as high-scoring mutations in other genes) combined together with truncating mutations are primary candidates for cancer drivers.

One can also notice that the non-uniformity of mutation distribution in non-cancer genes is outstandingly high for truncating and missense mutations in breast and kidney cancer; this means that mutations in the top non-cancer genes mutated in these cancers are selected in tumor evolution. These new genes are not yet included in the list of cancer genes. - However, we do not report these genes in this study, because the main purpose of this work is to study the ability of the evolutionarily derived FIS to recognize selection in cancer, rather than analysis of the roles of the specific genes in breast and kidney cancers. The detail analysis of cancer specific genes should be done in a separate study.

Table S3A. The effective numbers of genes derived from distributions of predicted functional and truncating mutations.

| **Cancer** | **Genes (MM+TM)** | **Genes eff (MM+TM)** | **Genes (FIS>2.5+TM)** | **Genes eff (FIS>2.5+TM)** | **CanGen**  **(MM + TM)** | **CanGen eff (MM + TM)** | | **CanGen (FIS>2.5 + TM)** | | **CanGen eff (FIS>2.5 + TM)** | |  |
| --- | --- | --- | --- | --- | --- | --- | --- | --- | --- | --- | --- | --- |
| Ovarian | **7578/4646** | 1358/814 | 3459/527 | **232/49** | 846/498 | | 40/16 | | 417/69 | | **10/1** | |
| Lung | **12089/5866** | 4336/1647 | 7402/1179 | **3089/196** | 1196/478 | | 209/70 | | 834/116 | | **156/4** | |
| Kidney | **9205/4411** | 2405/882 | 5386/592 | **954/57** | 963/403 | | 93/31 | | 637/77 | | **37/0** | |
| Brain | **5446/2903** | 1211/489 | 2769/226 | **346/16** | 652/247 | | 61/4 | | 420/15 | | **30/0** | |
| Breast | **9835/5059** | 3174/1362 | 5465/689 | **1184/75** | 1015/448 | | 126/31 | | 648/81 | | **54/1** | |
| Colon | **6554/3470** | 1511/629 | 3371/287 | **409/20** | 731/345 | | 58/11 | | 421/35 | | **22/0** | |

Two numbers in each cell present (i) a total number of affected genes and (ii) a number of genes enriched by low functional mutations (potential “passengers”); these numbers are used in building histograms of Fig.3). The effective numbers of genes are computed by Eqs.3-6; because of selection, predicted functional mutations are distributed non-uniformly across genes and a significant fraction of all mutations can be represented by a relatively small fraction of all genes, defined as the effective number of genes. Numbers of the effective genes can be used as the estimates of the total number of genes under selection pressure.

Table S3B. Percentages of genes enriched by low functional mutations (potential “passengers”).

| **Cancer** | **Genes (MM+TM)** | **Genes eff (MM+TM)** | **Genes (FIS>2.5+TM)** | **Genes eff (FIS>2.5+TM)** | **CanGen (MM + TM)** | **CanGen eff (MM + TM)** | **CanGen (FIS>2.5 + TM)** | **CanGen eff (FIS>2.5 + TM)** |
| --- | --- | --- | --- | --- | --- | --- | --- | --- |
| Ovarian | 61 | 60 | 15 | 21 | 59 | 40 | 17 | 10 |
| Lung | 49 | 38 | 16 | 6 | 40 | 33 | 14 | 3 |
| Kidney | 48 | 37 | 11 | 6 | 42 | 33 | 12 | 0 |
| Brain | 53 | 40 | 8 | 5 | 38 | 7 | 4 | 0 |
| Breast | 51 | 43 | 13 | 6 | 44 | 25 | 13 | 2 |
| Colon | 53 | 42 | 9 | 5 | 47 | 19 | 8 | 0 |
| Average | **53** | **43** | **12** | **8** | **45** | **26** | **11** | **2** |

The percentages of potential “passenger” genes presented in the histograms of Fig.4. Potential “passengers” genes are defined as genes that have more or equal number of low functional mutations (FIS<1.0) as compared to a number of high-functional mutations, i.e. missense mutations of FIS>2.5 and truncating mutations; “MM+TM” refers to genes affected by either missense (MM; FIS>-4) or truncating (TM) mutations; CanGenes stands for “cancer genes”. Note the systematic reduction of the percentage of genes enriched by low functional mutations for transitions from “Genes” to “Genes eff” and also for transition from “MM+TM” to (FIS>2.5+TM).
